# Fused expression of Sm1-Chit42 proteins for synergistic mycoparasitic response of *Trichoderma afroharzianum* on *Botrytis cinerea*

**DOI:** 10.1186/s12934-023-02151-w

**Published:** 2023-08-17

**Authors:** Hongyi Liu, Shaoqing Wang, Bo Lang, Yaqian Li, Xinhua Wang, Jie Chen

**Affiliations:** 1 School of Agriculture and Biology, Shanghai Jiao Tong University, Shanghai, China; 2 State Key Laboratory of Microbial Metabolism, Shanghai Jiao Tong University, Shanghai, China

**Keywords:** *Botrytis cinerea*, Chitin degradation, Hyphae recognition, Mycoparasitism, *Trichoderma afroharzianum*

## Abstract

Sm1 and Chit42 of *Trichoderma* have been universally confirmed as crucial biocontrol factors against pathogen infection through induced resistance and mycoparasitism, respectively. However, not enough work has been conducted to understand the novel function of fused expression of these two proteins in *Trichoderma.* The results of this study demonstrated that Sm1-Chit42 protein (SCf) engineered *T. afroharzianum* strain *OE:SCf* exerted synergistic inhibition to *Botrytis cinerea* growth at multiple stages of mycoparasitic interaction of *T. afroharzianum* and *B. cinerea* including chemotropism sensing, hyphal coiling, hydrophobicity modulation, cell wall adhesion, virulence reduction and pathogen killing by ROS. These results highlight a novel mycoparasitic system in *Trichoderma* strains engineered with Sm1-Chit42 chimeric protein to combat *B. cinerea* growth and reproduction, which would lay a strong foundation for exploring a new engineered *Trichoderma* biofungicide created with chimeric proteins in the future.

**Supplementary Information:**

The online version contains supplementary material available at 10.1186/s12934-023-02151-w.

## Introduction

Microbial interactions are ubiquitous and versatile, ranging from mutualism to parasitism and competition. Fungi have numerous mechanisms to communicate with other organisms, including plants, animals, and other microorganisms [1]. Interestingly, most fungal relations are competitive or combative [1, 2]. *Trichoderma* is one of the most widely used biocontrol fungi and is usually viewed as a green alternative to chemical pesticides in agriculture, and it has successfully been used to control a range of plant diseases in recent decades [3–5]. Mycoparasitic *Trichoderma* species attack and parasitize various phytopathogenic fungi, such as *Magnaporthe oryzae*, *Botrytis cinerea*, and *Fusarium graminearum* [6–10].

*Trichoderma* and phytopathogenic fungi interactions are typical fungus-fungus wars, which are usually associated with mycoparasitism and competition by the secretion of antibiotics, peptides, and cell wall-degrading enzymes [11–13]. The secreted Sm1 protein belongs to the cerato-platanin protein family, which consists of small secreted proteins that are abundantly produced by filamentous fungi with all types of lifestyles [14]. The Cerato-Platanin protein Epl-1 (Sm1) from *T. harzianum* is involved in mycoparasitism, and plant resistance induction [15, 16]. In addition, in the mycoparasitic *Trichoderma* spp., Sm1 evolved the ability to modify the hydrophobicity of the fungal hypersphere and, thus, facilitate the nutritional versatility of *Trichoderma* [17, 18].

In the case of mycoparasitic *Trichoderma*, the fungal pathogen cell wall is exposed to the action of *Trichoderma* hydrolases such as chitinases, which digest the fungal cell wall chitin as an antifungal mechanism and release chitin oligomers that can be recognized by the host plant [19–21]. Fungal chitinases are therefore not only involved in exogenous chitin decomposition but also fungal cell wall degradation and remodeling [22, 23]. Chitinases have diverse sources, characteristics, and mechanisms, which present challenges to the development of optimization procedures and standardization techniques for enhancing practical applications. With the development of protein engineering techniques, these difficulties can be overcome by modifying or redesigning chitinases to improve specific features required for specific applications [24]. Fungal chitinases consist of glycoside hydrolases 18 (GH 18), GH 19, and GH 20 modules; thus, they also include different carbohydrate-binding modules (CBMs) [25, 26]. Chitinase 42 (Mchit42) of *Metarhizium anisopliae* has been proven to be a major contributor to the antagonistic activity against *B. cinerea* [27] however, MaChit42 lacks CBM. The Sm1-like proteins have been proven to bind chitin [18, 28–30]. It was speculated that the integration of Sm1 and Chit42 proteins may allow Sm1 as the CBM of Chit42 to complement the functions of Chit42, thereby potentially developing a synergistic mechanism against pathogenic infection. Therefore, constructing chimeric proteins from two different effectors through a linker has become a new strategy for *Trichoderma* to improve its biocontrol activity against fungal phytopathogen infection. This study was designed to elucidate the mechanism by which engineered *Trichoderma* with the chimeric proteins Sm1-Chit42 confers synergistic control of *B. cinerea*.

## Materials and methods

### Strains, plasmids, and culture conditions

**Fig. 1:**
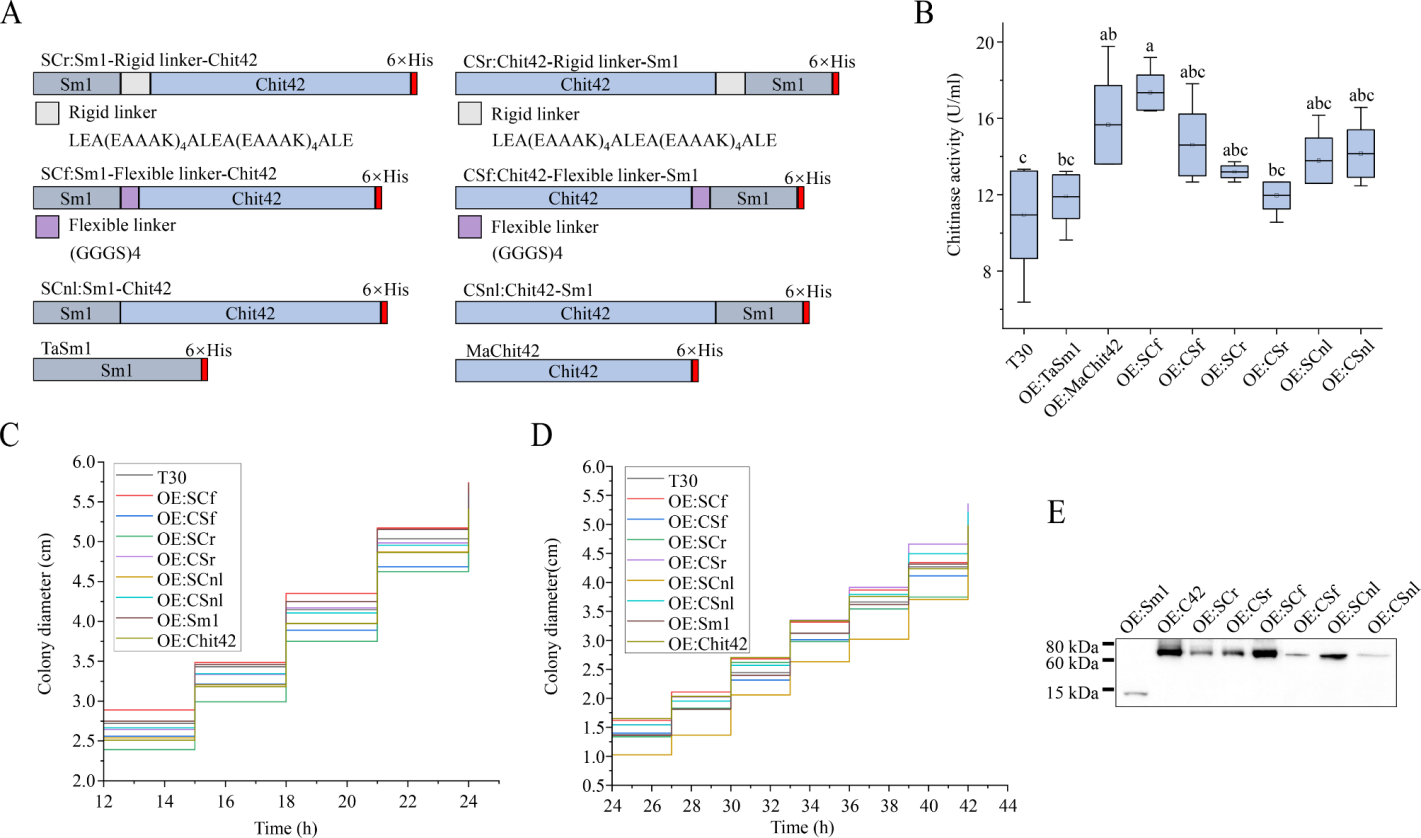
Construction and enzyme activity of *T*. *afroharzianum* engineered strains. (**A**) Illustration of open reading frame of chimeric protein; (**B**) Chitinase activities of *T. afroharzianum* wild-type (T30) and engineered strains. The chitinase activity of *OE:SCf* was higher than that of other engineered strains and wild-type (T30); Diameter of mycelium growth of *T. afroharzianum* engineered strains cultured in PDA medium with different inoculation methods of the colony plugs (**C**) and spore suspension (**D**); (**E**) Western blot analysis of chimeric protein in the culture broth of *T. afroharzianum*. Letters represent conditions with significant differences according to the post hoc ANOVA Fisher’s test (p < 0.05)

*E. coli* DH5α was used for molecular cloning and plasmid construction, while *Trichoderma afroharzianum* CGMCC22479 (China General Microbiological Culture Collection Center) served as the expression host for *MaChit42*, *TaSm1*, *SCf*, *CSf*, *SCr*, *CSr*, *SCnl* and *CSnl* (S: *TaSm1*; C: *MaChit42*; f: flexible linker; r: rigid linker; nl: none-linker, Fig. 1A), while the pCambia-1300 was used as the *Trichoderma* overexpression vector. All the bacterial strains were cultured in Luria-Bertani (LB) medium at 37 °C and 220 rpm. Fungal phytopathogenic strains used in this study were collected by Shanghai Jiaotong University, Institute of Agricultural Environmental Microbial Engineering, including *B. cinerea* (Accession number:MN420829), *M. oryzae* (Accession number: GCA_002368485.1), *F. graminearum* (Accession number: MN396567), *F. verticillioides*[31], *Rhizoctonia solani* (Accession number: MN422011), *Bipolaris maydis* [32], *Metarhizium anisopliae*[33], and *Curvularia lunata* (Accession number: GCA_000743335.1) were routinely grown on potato dextrose agar (PDA) plate at 28 ℃.

### Plasmid construction and *trichoderma afroharzianum* transformation

The primers and linker types used in this study are listed in Table S1. The genes encoding *MaChit42* from *Metarhizium anisopliae* (GenBank: DQ011865.1) and *TaSm1* from *Trichoderma afroharzianum* (GenBank: XM_024919062.1). The plasmid backbone of pCambia-1300 including the promoter trpC and terminator trpC is from our laboratory. Sequences of *MaChit42*, *TaSm1*, *SCf*, *CSf*, *SCr*, *CSr*, *SCnl*, and *CSnl* were obtained by overlap PCR, both with a C-terminal his-tag. All the DNA fragments were amplified using Phanta Max Master Mix polymerase (Vazyme, Nanjing, China). Construction of the recombinant plasmids was entirely done by the operating instructions of the ClonExpress MultiS One Step Cloning Kit (Vazyme, Nanjing, China). The *Agrobacterium tumefaciens*-mediated transformation (ATMT) techniques were described in the literature [34]. Based on the resistance markers on the plasmids, the transformants of *Trichoderma* were selected on CYA (0.2% NNO_3_, 0.1% KHPO_4_·3H_2_O, 0.05% Kl, 0.05% MSO_4_·7H_2_O, 0.001% FSO_4_·7H_2_O, 3% Scrose) agar plates containing 300 µg/mL timentin and 200 µg/mL hygromycin. The engineered strains of *T. afroharzianum* were obtained by single spore separation.

### Interaction assays between engineered strains of *T. afroharzianum* and fungal phytopathogens

The *Trichoderma* and phytopathogens interaction were assessed by dual confrontation assays [17]. Agar plugs of fresh engineered strains and the plant fungal pathogens were pregrown on PDA at 25 °C for 4 days, and were placed on opposite poles of a PDA plate. Images of each plate were recorded by an MF image system (Shineso, Hangzhou, China) after incubation at 25 °C in darkness for 4–7 days. Fungal biomass was collected from the interacting and non-interaction sides after connection for 24 h, which could be used to determine the expression levels of mycoparasitism and signal transition-related genes. A quick method for visualizing the interaction process of engineered strains and *B. cinerea* was accomplished by using FM4-64 (AAT Bioquest, Pleasanton, USA) and CDCFDA (AAT Bioquest, Pleasanton, California) probe and Leica TCS SPS-II (Wetzlar, Germany) confocal laser scanning microscopy. The engineered strains and *B. cinerea* were cultured on a PDA plate containing glass paper. Glass paper plugs of *B. cinerea* were incubated with PBS buffer (137 mM NaCl, 2.7 mM KCl, 10 mM Na_2_HPO_4_, 2 mM KH_2_PO4, pH = 7.2) diluted 2–20 µM FM4-64 probe, and which were kept on ice for 15 min. The glass plugs were put on the PDA plate after twice PBS washing. Glass paper plugs of engineered strains were incubated at 28 ℃ with PBS buffer diluted 0.5 ~ 25 µM CDCFDA probe for 15 min, and then incubate at 37 ℃ for 30 min after changing the 37 ℃ preheated CDCFDA probe. The glass plugs of engineered strains were put on the PDA plate close to *B. cinerea* after twice PBS washing. The interaction process of engineered strains and *B. cinerea* was observed on confocal laser scanning microscopy after 12 h.

### Secretion assay

To perform assays of Sm1, Chit42, and chimeric protein secretion, the *T. afrohazianum* overexpression strains were cultivated in Czapek–Dox broth (3% Sucrose, 0.001% FeSO_4_ · 7 H_2_O, 0.05% MgSO_4_·7 H_2_O, 0.05% KCl, 0.1% K_2_HPO_4_, and 0.3% NaNO_3_). Secretion analysis was performed as described. Briefly, 7-day-old engineered strains culture supernatants (1 L culture supernatant per strain) were collected and clarified by filtration through a 0.22-µm polyvinylidene fluoride Millipore membrane (Merck, Darmstadt, Germany) respectively. Proteins were concentrated in equilibration buffer (50 mM Tris-HCl, pH = 7.4, 150 mM NaCl, 1 mM EDTA, 1% NP-40, 1 mM PMSF) by Millipore Amicon® Ultra (Merck). The resuspended proteins were analyzed by immunoblotting with mouse anti-His (YEASEN, Shanghai, China). As a control, the total proteins were extracted from wild-type mycelia.

### Quantification of *T. afroharzianum* chemotropism

**Fig. 2:**
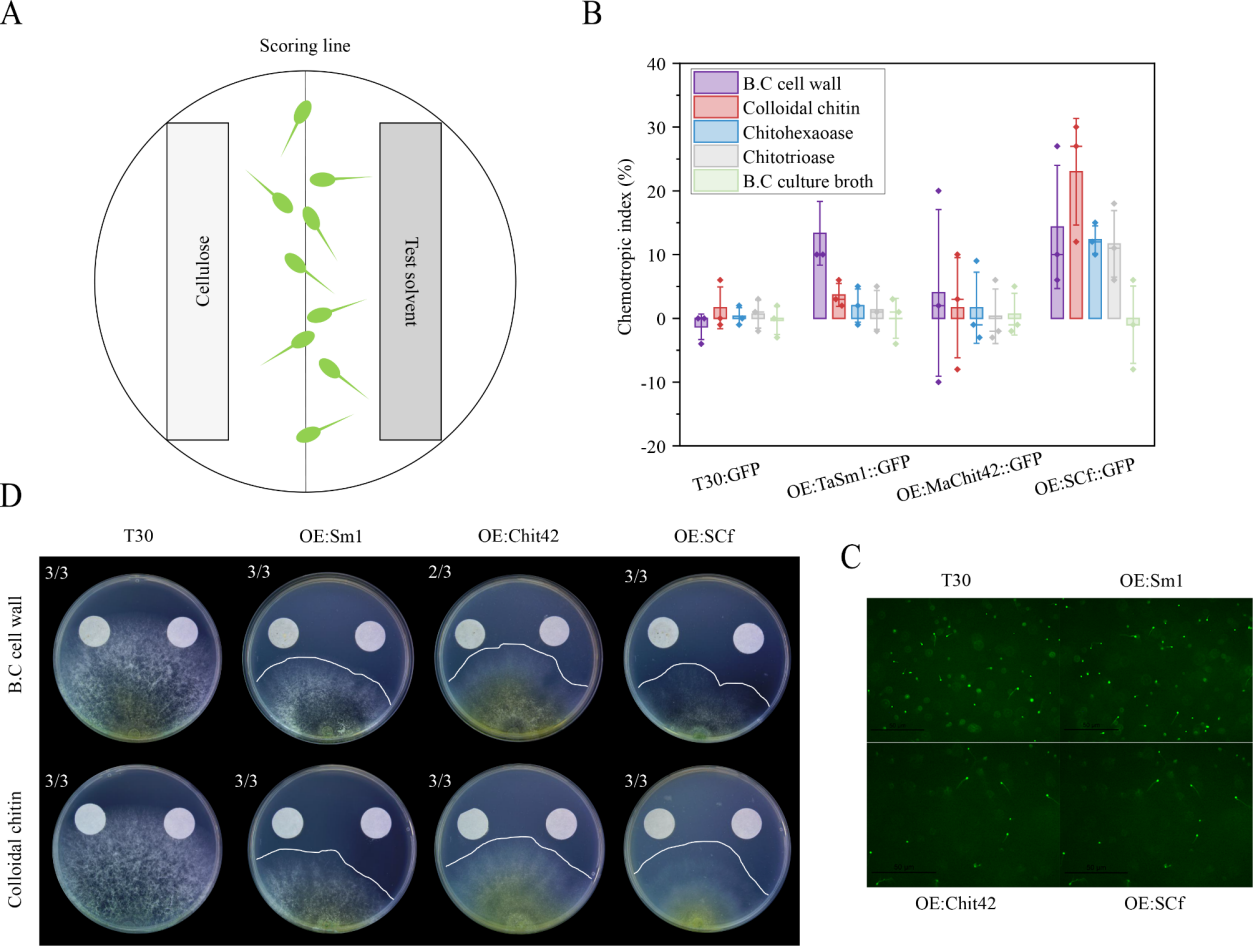
Chemotropism of *T. afroharzianum* engineered strains. (**A**) Schematic representation of the plate chemotropism assay. Test compound and solvent control are applied to opposite sides of a Petri dish containing a layer of water agar with 2 × 10^5^ /ml conidia spore of *T. afroharzianum* wild-type (T30), *OE:Sm1*, *OE:Chit42*, and *OE:SCf* at a distance of 0.5 cm from the central scoring line; (**B**) Directed growth of germ tubes after exposure to the indicated compounds. Letters represent conditions with significant differences according to the post hoc ANOVA Fisher’s test (p < 0.05); (**C**) Germination of conidia spore of *T. afroharzianum* over time. Germ tube emergence sites are visualized by inserted the GFP protein into *T. afroharzianum* wild-type (T30), *OE:Sm1*, *OE:Chit42*, and *OE:SCf*. DIC, differential interference contrast. Scale bar, 50 µm; (**D**) Mycelium chemotropism of *T. afroharzianum* towards cellulose (control solvent), *B. cinerea* cell wall, and colloidal chitin. The left filter slice is *B. cinerea* cell wall or colloidal chitin; The right filter slice is cellulose

This experiment is modified according to the method of Turrà et al. [35]. 2 mL of sterile water was added to the mature culture plates of *T. afroharzianum* wild-type and engineered strains (*OE:Sm1*, *OE:Chit42*, *OE:SCf*), the conidial suspensions were filtered through a sterile glass funnel and gauze into a sterile 15 mL falcon tube. Freshly obtained conidial spores of *T. afroharzianum* wild-type and engineered strains (*OE:Sm1*, *OE:Chit42*, *OE:SCf*) were embedded in 5 ml water agar (WA; 1%, w/v) (Oxoid, Thermo Fisher, MA, USA) at a final concentration of 2 × 10^5^ /ml and poured into a standard Petri dish (Fig. 2A). A central scoring line was drawn on the bottom of the plate, and two parallel wells were cut into the WA layer on both sides at 5 mm distance from the scoring line. Then, 50 µl of the test compound solution or the solvent control was added to the wells at both sides of the scoring line. In gradient competition experiments, solutions of the two different test compounds were applied at both sides of the scoring line. Tested compounds and standard concentrations were: chitohexaose and chitotriose at 50 mM; or cellulose, *B. cinerea* cell wall, and colloidal chitin, all at 1% (w/v); or unconcentrated *B. cinerea* culture broth. Cellulose was used as solvent control. To measure the chemotropism of *T. afroharzianum* towards test compounds, plates were maintained in a plastic box at 28 °C in the dark for the indicated periods (13 h unless otherwise stated). Chemotropism of conidial germ tubes was quantified with a Leica binocular microscope (200 × magnification), by counting the number of hyphal tips pointing toward the test compound and those pointing toward the solvent control. The chemotropic index was calculated as Eq. (1):

1 $$chemotropic index\left(\%\right)=\frac{{H}_{test}-{H}_{solvent}}{{H}_{total}}\times 100$$

where ${H}_{test}$is the number of hyphae growing towards the test compound, ${H}_{solvent}$ is the number of hyphae growing towards the solvent control, and ${H}_{total}$ is the total number of hyphae counted. For each test compound, a total of 100 hyphal tips were scored. All experiments were performed at least twice. Statistical analysis was conducted using a t-test.

### Chitinase activity assay

Chitinase activity was measured using the Micro Chitinase Assay Kit (SolarBio, China) following the manufacturer’s instructions. The *T. afrohazianum* overexpression strains were cultivated in Czapek–Dox medium. Secretion analysis was performed as described. Briefly, 7-day-old engineered strains culture supernatants (1 mL culture supernatant per sample) were collected. The chitinase activities can be measured fluorometrically at a wavelength of 585 nm. Measurements were done by incubating 100 ng of recombinant protein for 60 min at 37 °C. Fluorescence was detected with a Microplate Reader (SpectraMaxi3x, Molecular device) at room temperature. Each measurement was performed in triplicate and experiments were repeated at least four times with independent batches of recombinant proteins. Each treatment was replicated three times.

### Gene expression analyses by qPCR

The transcript levels of the *TaSm1* gene were determined by qPCR in *T. afroharzianum* engineered strains. And the transcript levels of mycoparasitism and signal transition-related genes were determined in the interaction process of engineered strains (Wild-type, OE:Sm1, OE:Chit42, OE:SCf) and *B. cinerea*. Transcript levels of *B. cinerea* genes affected by *T. afroharzianum OE:SCf* engineered strain were determined by qPCR. For quantification of gene expression, total RNAs were extracted from different tissues using the TRIzol reagent according to the manufacturer’s instructions (Invitrogen, USA). 1 µg RNA was reverse-transcribed into cDNA using HiScript III RT SuperMix for qPCR (Vazyme). The cDNAs were used as templates for PCR with gene-specific primers (Table S1). Quantitative real-time RT-PCR analysis (qRT-PCR) was performed using ChamQ Universal SYBR qPCR Master Mix (Vazyme) on Roche lightcycler96 (Basel, Switzerland). All experiments were repeated three times independently.

### Quantification and statistical analysis

All statistical analyses described in this study were performed using Origin 2022b. The number of replicates for each experiment is reported in the figure legends. For the chitinase activity assays, WCA, and qPCR, statistical comparison among groups was performed by one-way ANOVA with the Fisher’s Least Significant Difference (LSD) post hoc test.

## Results

### Construction of engineered *T. afroharzianum* strains with Sm1-Chit42

An expression vector harboring a copy of the *Sm1*-*Chit42* gene with its trpC terminator under the control of the trpC promoter from *Aspergillus nidulans* was used to overexpress *Sm1*-*Chit42* in *T. afroharzianum. MaChit42*, *TaSm1*, *SCf*, *CSf*, *SCr*, *CSr*, *SCnl*, and *CSnl* (Fig. 1A, Figure S1A-D) overexpression vectors were constructed by using the same method. At least 15 putative transformants of each of *T. afroharzianum* engineered strains were found to have the expected overexpression cassette. *OE:MaChit42-6*, *OE:TaSm1-1*, *OE:SCf-7*, *OE:CSf-11*, *OE:SCr-5*, *OE:CSr-3*, *OE:SCnl-2* and *OE:CSnl-7* transformants of *T. afroharzianum* were identified as single copy strains by using Southern blotting (Figure S1E). Therefore, the engineered strains were designated *MaChit42*, *TaSm1*, *SCf*, *CSf*, *SCr*, *CSr*, *SCnl*, and *CSnl* by removing the number mark in further studies. The expression of the *Sm1* gene in the above mutants was detected by qPCR. The results showed that the expression level of Sm1 in *OE:SCf* strain remained high in PDA and PD, and the Sm1 expression of other engineered strains on the PDA plate was lower than *OE:SCf* (Figure S1F). As MaChit42 and TaSm1 proteins are similarly secreted from *M. anisopliae* and *T. afroharzianum* respectively, six chimeric protein engineered strains were selected for further verification by Western Blot analysis (Fig. 1E), and the results indicated that chimeric proteins are targeted to the extracellular space.

### Chitinase activities of Sm1-Chit42 engineered *T. afroharzainum* strains

The chitinase activity (U/ml) of wild-type (T30) and engineered strains with colloidal chitin as substrate were T30 (10.95 ± 3.96), *OE:TaSm1* (11.90 ± 1.98), *OE:MaChit42* (15.66 ± 3.56), *OE:SCf* (17.34 ± 1.59), *OE:CSf* (14.61 ± 2.80), *OE:SCr* (13.21 ± 0.53), *OE:CSr* (11.97 ± 1.22), *OE:SCnl* (13.78 ± 2.06), and *OE:CSnl* (14.16 ± 2.15) after concentration (Fig. 1B). The chitinase activity of *OE:SCf* was higher than that of other engineered strains, which was a significant (*P* < 0.05) increase of 58.36% compared to the wild-type (T30).

### Chimeric protein offers synergistic inhibitory effects in *trichoderma* against phytopathogenic fungi

**Fig. 3:**
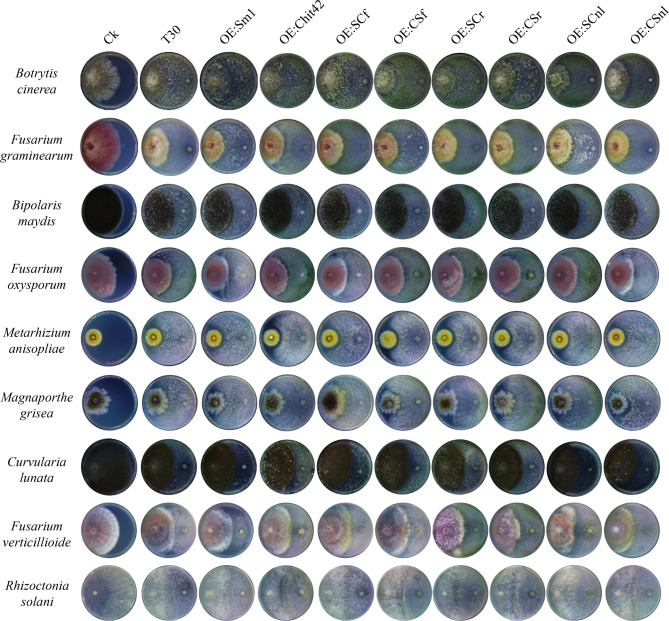
Antagonistic activity of *T. afroharzianum* engineered strains against nine important plant pathogenic fungi. The radius of the interaction zone (centimeter, cm) was measured for each dual confrontation of *T. afroharzianum* engineered strains with *B. cinerea*, *M. oryzae*, *F. graminearum*, *F. oxysporum, F. verticillioides*, *R. solani*, *B. maydis*, *M. anisopliae*, and *C. lunata*

A dual confrontation test of the engineered *T. afroharzianum* strains against multiple plant fungal pathogens (including *B. cinerea*, *M. oryzae*, *F. graminearum*, *F. oxysporum, F. verticillioides*, *R. solani*, *B. maydis*, *M. anisopliae*, and *C. lunata*) was performed as shown in Fig. 3. No significant morphological difference was observed due to the overexpression of chimeric proteins, except that the *OE:SCf* engineered strains of *T. afroharzianum* showed a higher growth rate than the other engineered strains (Fig. 1C-D). Overexpression of chimeric protein in the *Trichoderma* strain showed different antagonism against different pathogens (Table 1), and the inhibitory effects of *OE:SCr* and *OE:CSr* were 74.38 ± 4.88 and 70.00 ± 26.46 respectively. However, the inhibitory effect of these two strains was lower than that of *OE:MaChit42* (91.33 ± 15.01). The overexpression of SCf chimeric protein enhanced antagonism against *B. cinerea* (80.00 ± 4.10), *F. oxysporum* (44.73 ± 11.26), *F*. *verticillioides* (48.65 ± 3.33), and *R. solani* (86.98 ± 11.30) compared to that of the wild-type (T30), *OE:TaSm1* and *OE:MaChit42* strains. However, the antagonistic effect of *OE:SCf* on *F. oxysporum* and *F*. *verticillioides* was not significantly different (*P* > 0.05) from that of wild-type (T30) strains. In addition, none of the engineered strains showed obvious antagonism to *M. anisopliae*, and overexpression of fusion did not affect the self-recognition of *MaChit42* on *M. anisopliae* cell wall.

**Table 1:** Inhibitory effects of *T. afroharzianum* strains on phytopathogens

	Inhibitory rate (%)								
	T30	OE:SCf	OE:CSf	OE:SCr	OE:CSr	OE:SCnl	OE:CSnl	OE:Sm1	OE:Chit42
Botrytis cinerea	43.05 ± 2.84b	80.00 ± 4.10a	72.96 ± 18.63a	63.53 ± 21.44ab	74.85 ± 4.37a	75.81 ± 25.04a	60.38 ± 14.21ab	70.65 ± 8.66a	61.77 ± 3.80ab
Fusarium graminearum	51.23 ± 5.94bc	55.85 ± 5.77bc	50.64 ± 1.11bc	74.38 ± 4.88ab	70.00 ± 26.46abc	49.92 ± 5.89c	67.97 ± 27.73abc	57.26 ± 2.38bc	91.33 ± 15.01a
*Bipolaris* *maydis*	100	100	100	100	100	100	100	100	100
Fusarium oxysporum	37.29 ± 10.93ab	44.73 ± 11.26a	34.32 ± 9.69ab	26.74 ± 8.34b	36.23 ± 7.12ab	30.22 ± 7.36ab	44.63 ± 9.30a	39.93 ± 9.21ab	35.83 ± 7.03ab
Metarhizium anisopliae	13.14 ± 1.78ab	6.16 ± 5.33b	12.28 ± 8.46ab	15.04 ± 7.82ab	17.21 ± 4.89ab	14.03 ± 6.74ab	19.14 ± 4.20a	19.02 ± 9.10a	17.12 ± 7.36ab
Magnaporthe grisea	30.89 ± 11.81c	37.12 ± 9.92bc	43.04 ± 6.04abc	40.29 ± 8.62abc	49.94 ± 2.09a	40.66 ± 1.95abc	33.24 ± 5.24bc	44.26 ± 8.27ab	42.51 ± 6.19abc
*Curvularia* *lunata*	100	100	100	100	100	100	100	100	100
*Fusarium* *verticillioide*	38.79 ± 4.17ab	48.65 ± 3.33a	44.44 ± 2.23ab	39.50 ± 1.82ab	43.46 ± 8.83ab	44.57 ± 5.32ab	42.28 ± 1.40ab	29.80 ± 2.47b	34.60 ± 8.11ab
*Rhizoctonia* *solani*	61.46 ± 3.93b	86.98 ± 11.30a	59.89 ± 0.90b	58.85 ± 1.80bc	59.38 ± 2.71bc	58.33 ± 0.90bc	51.56 ± 4.13c	57.81 ± 4.6bc	65.63 ± 2.70b

Letters represent conditions with significant differences according to the post hoc ANOVA Fisher’s test (p < 0.05)

### SCf chimeric protein triggered early sensing in the interaction between *T. afroharzianum* engineered strain and *B. cinerea*

**Fig. 4:**
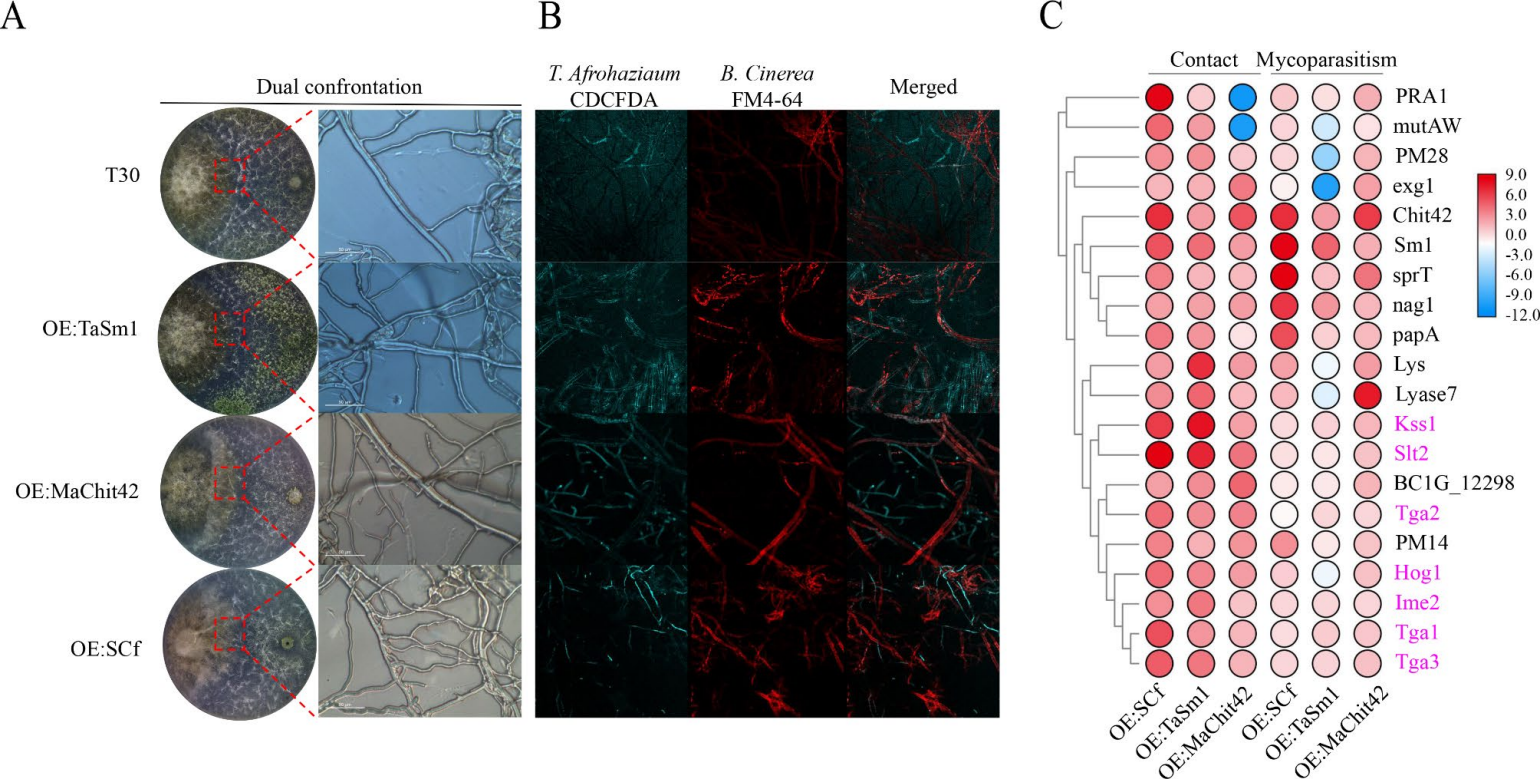
Dual confrontation assay between *T*. *afroharzianum* and *B. cinerea*. (**A**) Images of the fungal-fungal interaction between *T. afroharzianum* wild-type (T30), *OE:Sm1*, *OE:Chit42*, *OE:SCf* were recorded after inoculation on PDA (25°C in darkness) for 4–7 days; (**B**) Images of ROS burst of the fungal-fungal interaction between *T. afroharzianum* wild-type (T30), *OE:Sm1*, *OE:Chit42*, *OE:SCf* The *T. afroharzianum* strains were stained by CDCFDA and *B. cinerea* was stained by FM4-64. The hyphae coiling can be observed on *T. afroharzianum OE:SCf*; (**C**) Heatmap and cluster categorization of *T. afroharzianum* mycoparasitism-related genes expression when hyphae contact and after hyphae contact

Mutually sensitive sensing between biocontrol fungi and phytopathogenic fungi is usually an early response necessary for initiating a follow-up recognition and mycoparasitism [36]. The mycelium of *T. afroharzianum* wild-type, *OE:TaSm1*, *OE:MaChit42*, and *OE:SCf* strains (Fig. 4A) showed parallel hyphal growth around *B. cinerea* and no cell wall degradation debris was detected in the interaction zone. In the *T. afroharzianum* wild-type and *OE:MaChit42* strain interaction zone, a sparse mycelial mass attached to the mycelium of *B. cinerea* was observed with no hyphal degradation (Fig. 4A). In the *T. afroharzianum OE:TaSm1* and *OE:SCf* strain interaction zone, more mycelia adhered around the mycelium of *B. cinerea* with the preliminary hyphal degradation (Fig. 4A). The interaction between engineered *T. afroharzianum* and *B. cinerea* was also observed using confocal laser microscopy (Fig. 4B). The typical *Trichoderma* mycoparasitic action with hyphal coiling around the pathogen was observed only in the *T. afroharzianum OE:SCf* strain treatment but not in wild-type *T. afroharzianum* wild-type, *OE:TaSm1*, or *OE:MaChit42*. RT-qPCR results showed that the expression of *Sm1 gene* in the engineered strain was more significantly increased in the presence of *B. cinerea*, *M. oryzae*, *F. graminearum*, *F. oxysporum, F. verticillioides*, *R. solani*, *B. maydis*, *M. anisopliae*, and *C. lunata*, revealing that specific inductive response occurred between the engineered strains and *B. cinerea* (Figure S2). That implies that SCf chimeric protein triggered early sensing at the early mutual interaction stage.

### ROS release to kill pathogens in the interaction of the *T. afroharzianum OE:SCf* engineered strain and *B. cinerea*

ROS have been demonstrated to have dual functions in biocontrol (killing pathogens) and ISR to host plants. The CDCFDA fluorescence probe is also used to detect the ROS burst in the microorganism. Interestingly, the ROS burst could be observed in the mycelium of the *T. afroharzianum OE:SCf* strain in the mycoparasitic interaction between the *T. afroharzianum OE:SCf* strain and *B. cinerea* (Fig. 4B), implying that the higher ROS release level induced by the chimeric protein makes the engineered strain more active in killing pathogens or triggering plant immunity. Similar behavior also occurred in the interaction between *T. afroharzianum* strains and *R. solani* (data not shown).

### The SCf chimeric protein triggered mycoparasitism and signal transduction genes to modulate the interaction between *T. afroharzianum* engineered strain and *B. cinerea*

Confrontation assays were performed between *T. afroharzianum* wild-type, *OE:TaSm1*, *OE:MaChit42*, and *OE:SCf* strains and *B. cinerea*. Mycelial RNAs from the confrontation area were extracted from hyphal contact and mycoparasitism. The relative expression levels of *Sm1*, *Chit42*, and eleven mycoparasitism gene markers were analyzed to determine the effect of the *SCf* gene on the modulation of mycoparasitism gene expression (Fig. 4C). In the process of hyphal contact between *T. afroharzianum OE:TaSm1* and *B. cinerea*, nine genes were upregulated, of which *Sm1*, *Lys*, *Kss1*, and *Slt2* were prominent. Seven genes were upregulated, of which *BC1G_12298*, *exg1*, *Chit42*, *Tga1*, and *Slt2* were more prominent in the process of hyphal contact between *T. afroharzianum OE:MaChit42* and *B. cinerea*. Thirteen analyzed genes showed a marked difference in their expression levels, indicating *SCf* fusion gene altered the *T. afroharzianum* perception of *B. cinerea*. The expression levels of signal transduction genes were increased by hyphal contact of *T. afroharzianum* engineered strains and *B. cinerea*. The signal transduction genes of the *T. afroharzianum OE:TaSm1* and *OE:SCf* strains were better activated in the hyphal contact process, of which the *Kss1* and *Slt2* genes were more important in this process. Despite the relatively stable gene expressions with slight variations, significant differences were noted in the expression modulation of the transduction genes in the process of hyphal contact and mycoparasitism. The signal transduction genes play an important role in hyphae contact before the mycelium of *T. afroharzianum* engineered strains covered *B. cinerea*. In the confrontation between *T. afroharzianum* engineered strains and *B. cinerea*, the gene expression level of mycoparasitism genes was divided into 3 clusters according to their expression pattern: Group 1 (*PRA1*, *mutAW*, *PM28*, and *exg1*), Group 2 (*Chit42*, *Sm1*, *sprT*, *nag1*, and *papA*) and Group 3 (*Lys* and *Lyase7*). The expression levels of genes in Group 2 were higher when *T. afroharzianum OE:SCf* interacted with *B. cinerea.* The results described above demonstrate that the SCf genes can modulate the expression of mycoparasitism and signal transduction genes to improve the antagonistic effects on *B. cinerea*.

### SCf chimeric protein improved the chemotropism of the engineered *T. afroharzianum* strain for scavenging chitin fragments

**Fig. 5:**
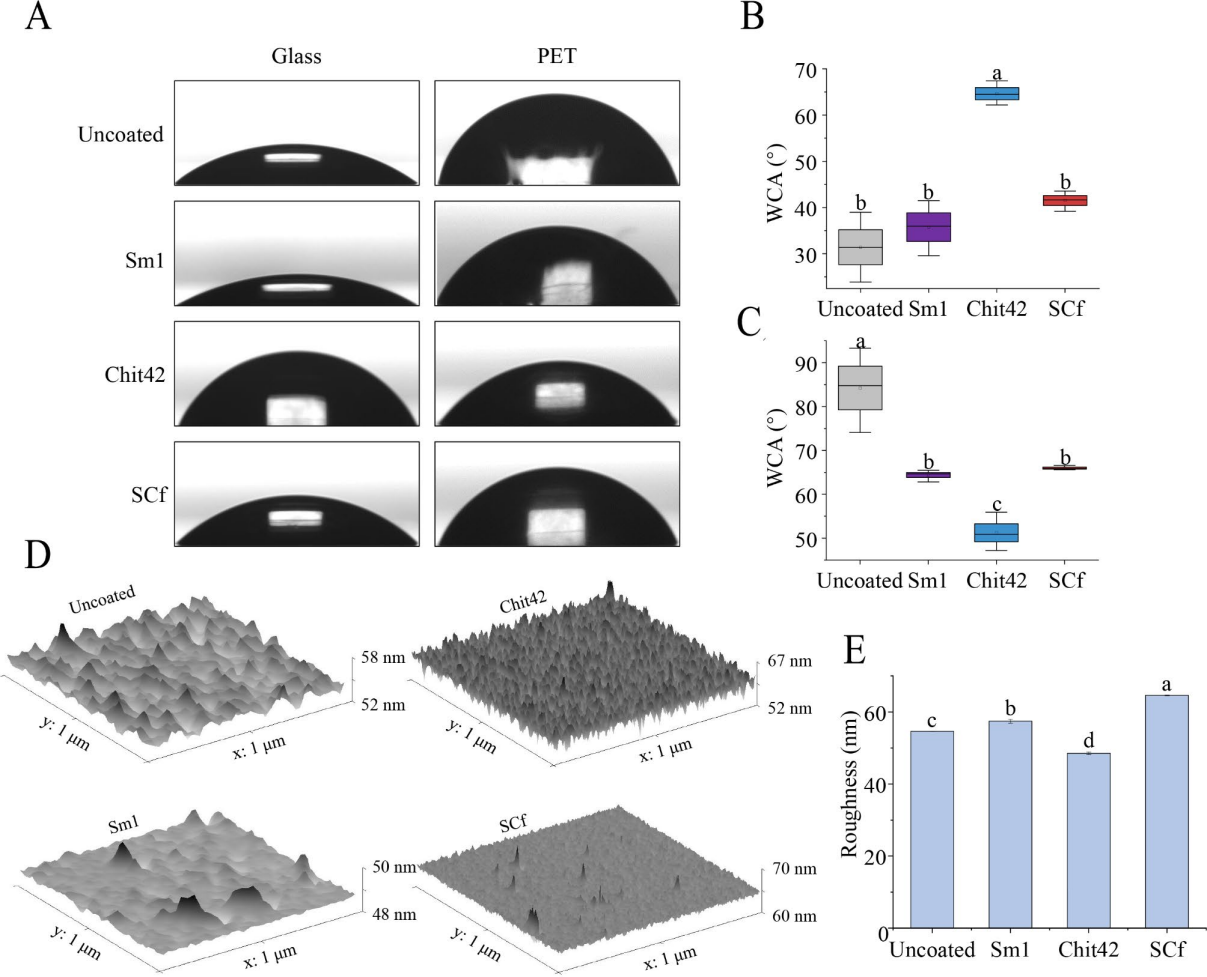
Cellular localization analysis of Sm1^FITC^., Chit42^FITC^, and SCf^FITC^ on hyphae of *B. cinerea*. FITC labeled protein was quantified by the Bradford method and 2.5 mΜ protein was added into the hyphae germinated from 600 µl *B. cinerea* mycelium pre-inoculated in a glass paper slide at 28 ℃ for 12 h. FM4-64 was used as *B. cinerea* cell membrane stains. The fluorescence intensity of FITC-labeled proteins and *B. cinerea* cell membrane were measured by ImageJ software

The chemotropism between *Trichoderma* and pathogenic fungi is also crucial for the successful contact of engineered strains over pathogens. In this study, we sought to understand whether chimeric protein in engineered stains cause faster growth toward chitin or derivatives from pathogen cell walls. First, we investigated what chemoattractants from *B. cinerea* sense chimeric proteins of engineered *T. afroharzianum* strains (Fig. 5B-C). The results showed that the *B. cinerea* cell wall is the chemoattractant for the pathogen to sense *T. afroharzianum OE:TaSm1 and OE:Chit42.* The order of the CI value of *T. afroharzianum OE:SCf* was colloidal chitin, *B. cinerea* cell wall, chitohexaose, chitotriose. The CI value of *B. cinerea* cell wall of *T. afroharzianum OE:SCf* was higher than *OE:TaSm1 and OE:Chit42.*The chitin derivates are the specific chemoattractants for *T. afroharzianum OE:SCf.* It was indicated that chitin and its derivatives are the chemoattractants for the pathogen to sense *T. afroharzianum OE:SCf*. The comparison of chemoattractants confirmed that the *B. cinerea* cell wall and colloidal chitin were the strongest chemoattractants. To evaluate the morphological responses of engineered *T. afroharzianum* strains to pathogen chemotropism, assays were conducted based on the changes in plate colonies. Distinct morphological responses to positive or negative chemotropic compounds were detected at this stage (Fig. 5D). The *B. cinerea* cell wall and colloidal chitin notably promoted colony extension of engineered strains, and the colony edge of *T. afroharzianum OE:Sm1* and *OE:SCf* grew more quickly toward the *B. cinerea* cell wall and colloidal chitin than the wild-type strain. Thus, the attraction of engineered strains to chitin and its derivatives in the pathogen make it more closely attached to the surface of the pathogen, thereby allowing *T. afroharzianum OE:SCf* to establish a stronger mycoparasitic relationship with the pathogen.

### SCf protein is secreted into the extracellular space to modulate surface hydrophobicity

**Fig. 6:**
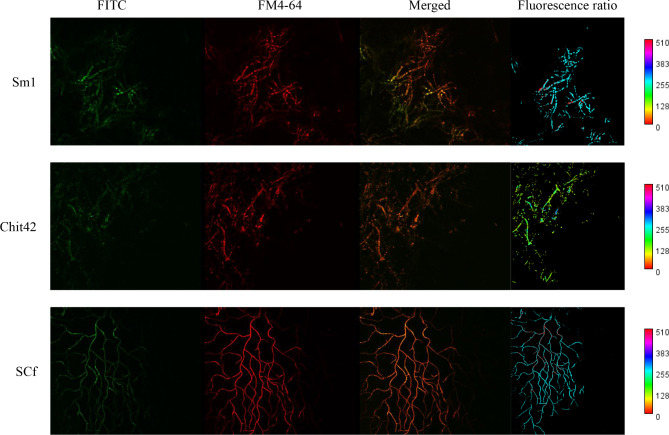
Hydrophobicity modulation ability of recombinant SCf. (**A**) Pictures of water droplets on recombinant Sm1, Chit42, and SCf coated PET and glass; (**B**) Contact angle measurements on glass slides; (**C**) Contact angle measurements on PET slides; (**D**) Atomic force microscopy of recombinant Sm1, Chit42, and SCf coated PET surfaces. Depicted are amplitude images of scans of 200 µm; (**E**) Roughness of recombinant Sm1, Chit42, and SCf coated PET surfaces determined by Gwyddion. Letters represent 532 conditions with significant differences according to the post hoc ANOVA Fisher’s test (p < 0.05)

The ability to modify the surface hydrophobicity of Sm1, Chit42, and SCf as surface-active proteins was illustrated by the results of the water contact angle (WCA) measurement with the proteins extracted from *T. afroharzianum* engineered strains. The data shown in Fig. 6A demonstrate the potential of recombinant Sm1, Chit42, and SCf protein to change the surface hydrophobicity of materials. Specifically, a coating of 50 µg/ml recombinant Sm1, Chit42, and SCf proteins on PET (hydrophobic) surface significantly reduced the surface hydrophobicity. However, the WCAs of the recombinant Sm1, and SCf proteins were 83.94° and 87° of the control, respectively, compared to the Chit42 protein, which was 67.64° of the control (Fig. 6B). The effect on glass (hydrophilic) was not significant (*P* **>** 0.05) (Fig. 6C). The hydrophobicity modulation ability of recombinant Sm1, Chit42, and SCf was investigated in more detail by AFM imaging. High-resolution imaging of the protein layers in PET with AFM revealed that SCf and Sm1 protein modulated rather irregular particle structures at concentrations of 50 µg/ml compared to sterile water and Chit42 protein (Fig. 6D). It was possible to measure the roughness of recombinant Sm1, Chit42, and SCf protein-treated PET surfaces by Gwyddion. Sm1 and SCf treated PET had a similar surface roughness (Fig. 6E), and the surface irregular particle height changed more than that of Chit42 and sterile water-treated PET. In addition, there was no significant difference in the effect of overexpressed *TaSm1*, *MaChit42*, and *SCf* on the hydrophilicity of the mycelia surface of engineered strains (Figure S3A-B). The hydrophobicity of the spore suspensions of *T. afroharzianum OE:TaSm1*, *OE:MaChit42*, and *OE:SCf* was not remarkably changed (Figure S3C-D). These results suggested that the SCf protein is secreted into the extracellular space to modulate the surface hydrophobicity of the host/pathogen.

### SCf chimeric protein can better adhere to the cell wall of *B. cinerea*

**Fig. 7:**
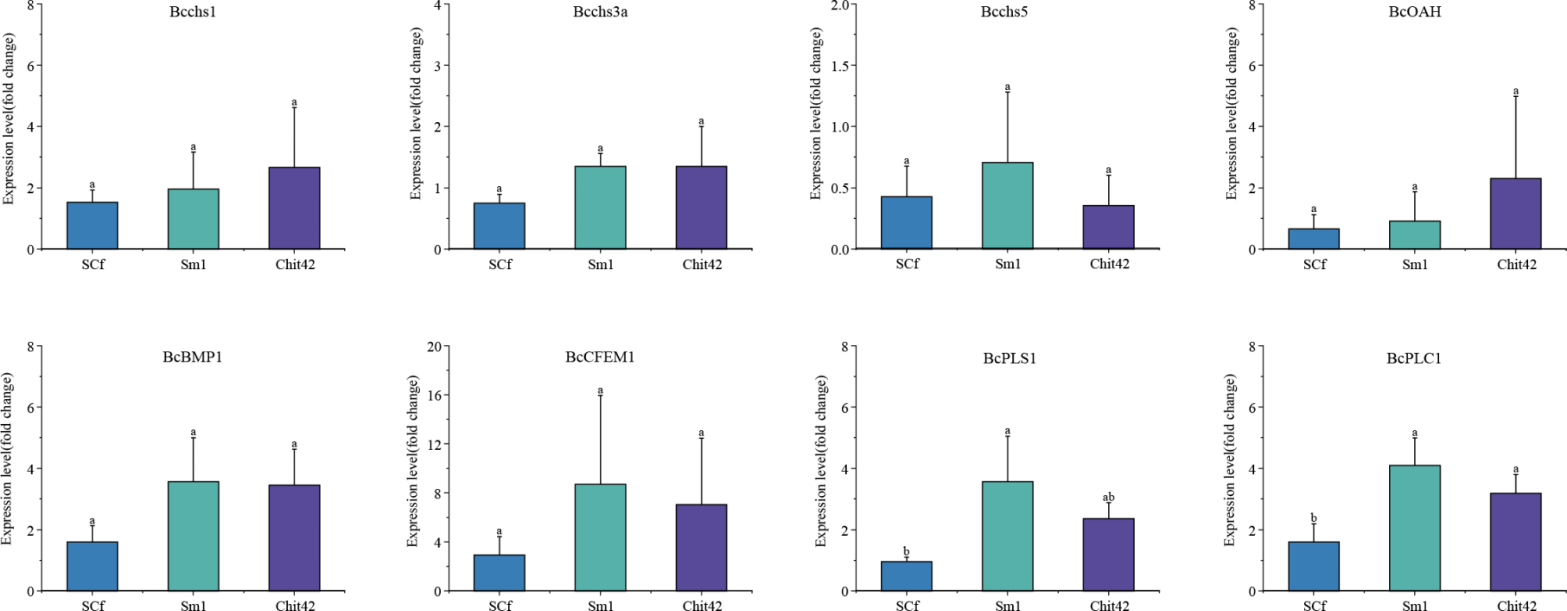
qPCR analysis of cell wall synthesis and virulence genes from *B. cinerea* during the interaction with *T. afroharzianum OE:Sm1*, *OE:Chi42*, and *OE:SCf*. Letters represent conditions with significant differences according to the post hoc ANOVA Fisher’s test (p < 0.05)

The Sm1, Chit42, and SCf proteins were labeled by FITC staining, and the mycelium of *B. cinerea* was stained by FM4-64 to observe the *B. cinerea* hyphal attachment ability of the Sm1, Chit42, and SCf proteins (Fig. 7). Confocal laser microscopy results showed that more Sm1^FITC^ and SCf^FITC^ were attached to *B. cinerea* hyphal than Chit42^FITC^ protein. The Sm1^FITC^ and SCf^FITC^ proteins showed more uniform adhesion to the surface of *B. cinerea* hyphae. This indicated that the chimeric protein improved the potential of *Trichoderma* to bind chitin and its derivatives from the pathogen compared to Chit42 protein.

### SCf fusion affected the expression of cell wall and virulence genes of *B. cinerea*

**Fig. 8:**
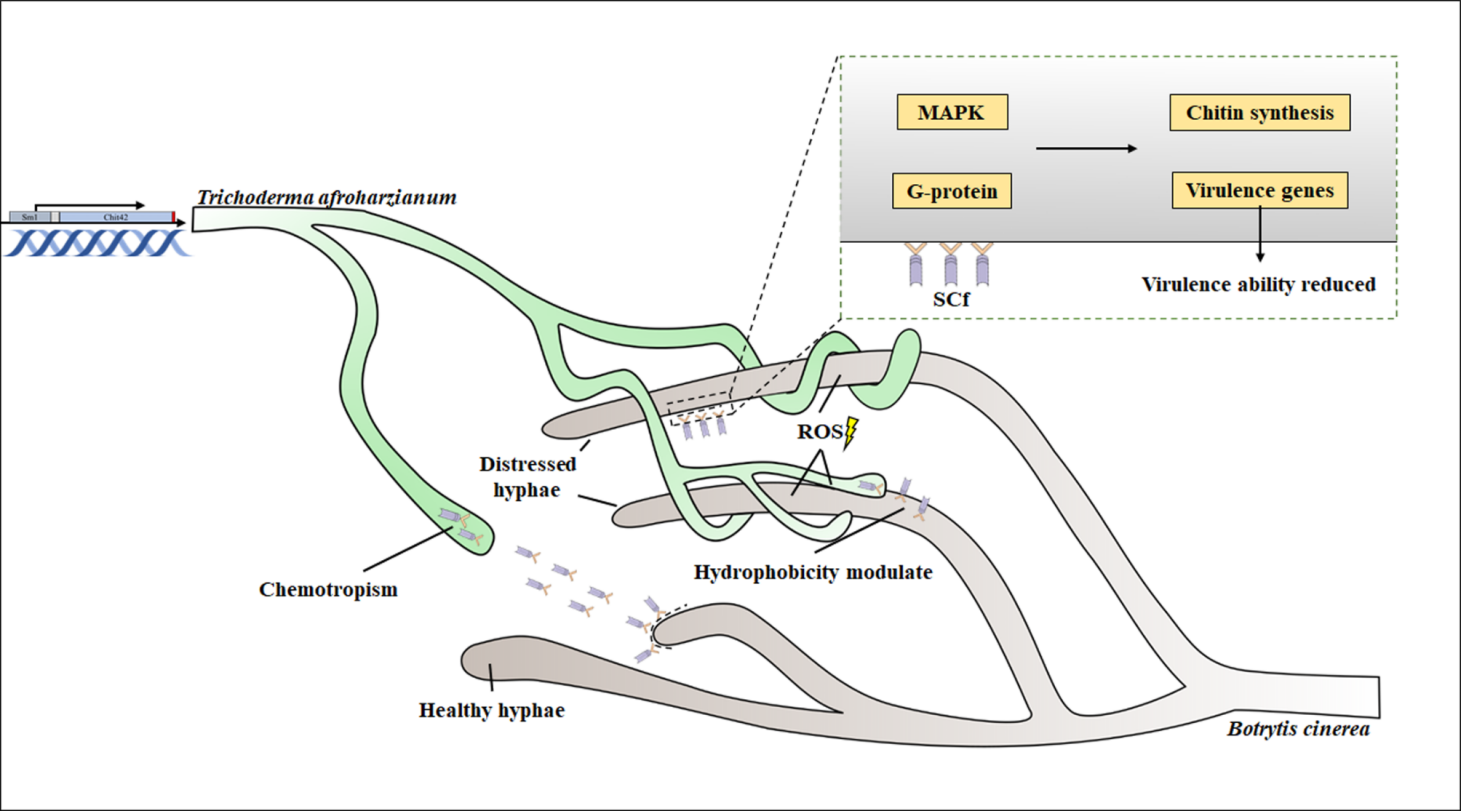
Mycoparasitic interaction model of *T. afroharzianum OE:SCf* with *B. cinerea.* The interaction was involved the chemotropism sensing, hyphae coiling, hydrophobicity modulation, cell wall adhere, virulence reduction, and killing pathogen by ROS

The expression levels of chitin synthesis (*Bcchs1*, *Bcchs3a*, and *Bcchs5*) and virulence-related genes (*BcOAH*, *BcCFEM*, *BcBMP1*, *BcPLC1*, and *BcPLS1*) in *B. cinerea* during the interaction with *T. afroharzianum OE:Sm1*, *OE:Chit42*, and *OE:SCf* strains are shown in Fig. 8. The *T. afroharzianum OE:SCf* strain more effectively inhibited the synthesis of chitin, however, there was no significant difference between the *T. afroharzianum* OE:Chit42 and OE:SCf strains. The expression levels of virulence-related genes (*BcOAH*, *BcCFEM*, *BcBMP1*, *BcPLC1*, and *BcPLS1*) were inhibited *T. afroharzianum OE:SCf* strain, even though the results showed no significant difference in *BcOAH*, *BcCFEM*, and *BcBMP1*. Overall, the expression of cell wall and virulence genes of *B. cinerea* was affected by the *T. afroharzianum OE:SCf* strain.

## Discussion

The biocontrol fungus *T. afroharzianum* is an avirulent symbiont with the ability to control plant disease through the production of antibiotic compounds, induction of plant resistance to pathogens, and mycoparasitism of other fungi [37–40], Mycoparasitism is a well-established mode of biocontrol microbes against fungal pathogens, for instance, *Trichoderma* uses the mechanism to offer antagonistic action against a group of fungal pathogens [41, 42].The mycoparasitism process conducted by *Trichoderma* involves chemotropism-based sensing, contact, and recognition, coiling and lysing of the target pathogen hyphae [43]. To enhance the biocontrol efficiency of *Trichoderma*, the construction of engineered *Trichoderma* strains with overexpressed functional genes is an alternative approach to improve *Trichoderma* agent performance in biological control [44]. Owing to their complementary advantages, Sm1 and Chit42 were linked and cotransformed into *T. afroharzianum* to develop a more powerful *Trichoderma* agent available to use for highly effectively parasitizing pathogens and inducing plant resistance to pathogen infection.

Chitin, one of the most widespread polysaccharides in nature, is a linear polymer of β-1-4-linked N-acetyl glucosamine (NAG) units [45]. This biopolymer is part of insect exoskeleton, crustacean shells, and fungal cell walls [46, 47]. *Trichoderma* chitinases degrade pathogen cell wall chitin components into low molecular weight chitooligomers, which have been demonstrated to induce local and systemic plant resistance [20, 48]. The chitinase activity of *OE:SCf* was higher than that of other engineered strains, showing a significant (P < 0.05) increase of 58.36% compared to that of the wild-type. The *T. afroharzianum OE:SCf* was proven to effectively control *B. cinerea* and *R. solani*. The chitinase from *Trichoderma* sp. drastically affected the cell walls of phytopathogens [48], indicating that the increase of chitinase activities can stimulate the antagonistic ability of the engineered *T. afroharzianum* strain.

The first evidence regarding the role of *SCf* genes in the *T. afroharzianum OE:SCf strain* indicates the generation of a sensing mechanism by developing more aerial hyphae to overgrow the *B. cinerea* aerial hyphae even though the sensing factors remain unclearly. Although positive chemotropism in response to prey-derived stimuli has been recognized as a key initiating feature of mycoparasitism early on [49], the cellular and molecular details of prey sensing are still poorly understood. Interspecies recognition in fungi has been linked to remote sensing of cell wall components [50–52]. It is generally accepted that fungi permanently secrete low amounts of extracellular chitinases that cause target fungi in the vicinity to release cell wall oligochitosan. Oligochitosan, in turn, is recognized as a ligand by the fungal mycoparasite and further induces the massive release of cell wall degrading enzymes from the mycoparasites [42, 53] promote degradation of the chitin in the target fungal pathogen cell wall. This cascade of events is considered the starting point for the activation of a mycoparasitic attack program in various *Trichoderma* species [50, 54]. To initiate the mycoparasitic process, early sensing or chemotropism plays a crucial role in the interaction of *Trichoderma* and fungal pathogens. In our study, *B. cinerea* cell wall and colloidal chitin were confirmed as the strongest chemoattractants for *T. afroharzianum OE:SCf*. Chemotropism between *Trichoderma* and fungal pathogens has been viewed as an important factor for effective mycoparasitism against fungal pathogens [55]. Interestingly, SCf from the engineered *Trichoderma* strain generated more activity to promote the growth of the engineered *Trichoderma* strain toward chitin and its derivatives from *B. cinerea*. In addition, the SCf protein is a complex hydrophobic protein that can modulate surface-surface hydrophobic contact between *Trichoderma and pathogens*, and between *Trichoderma* and plants, which would benefit the colonization of *Trichoderma* over pathogen mycelium and host plant. We assumed that the enhanced function of the SCf complex could be attributed to the changes in MaChit42 hydrophobicity resulting from partner Sm1 protein because Sm1 belongs to cerato-platanin (CP), a protein closely related to hydrophobin [18] Sm1 is characterized as an amphoteric protein membrane formed at the hydrophilic-hydrophobic interface, which can improve *Trichoderma* attachment on the surface of pathogenic fungus and plant leaf [18]. Therefore, we assumed that *OE:SCf* enhanced the destruction of the pathogen cell wall and induced plant resistance by secreting SCf protein into the extracellular space to modulate the surface hydrophobicity of the host/pathogen. After contact of *Trichoderma* mycelia on the surface of pathogen mycelia, the former typically coil around the latter and form of helix-shaped hyphae [42, 56], and this phenomenon is dependent on the recognition of lectins (CBM) from the fungal prey [57]. In our study SCf protein enhanced *Trichoderma* mycelial coiling around *B. cinerea* mycelium relative to the *Trichoderma* engineered strains with a single engineered protein.

SCf was demonstrated to promote reactive oxygen species (ROS) release from engineered *Trichoderma*, which was supposed to enhance the killing effects of pathogen or chitin-triggered immunity. Recently studies showed that *T. guizhouense* NJAU 4742 can use protease [58] and reactive oxygen species to kill pathogens such as *F. odoratissimum* (formerly known as FOC4) [2]. The key reactions of chitin biosynthesis are catalyzed by chitin synthase, a membrane-integrated glycosyltransferase that transfers GlcNAc from UDP-GlcNAc to a growing chitin chain [59–61]. Chitin synthase from the devastating soybean root rot pathogenic oomycete *Phytophthora sojae* (*PsChs1*) reveals the directional multistep mechanism of chitin biosynthesis and provides a structural basis for the inhibition of chitin synthesis. The homologous gene of *PsChs1* in *B. cinerea* was identified, however, the expression of *BcChs1* in interaction with *T. afroharzianum OE:SCf* was inhibited compared to that in interaction with *T. afroharzianum OE:Chit42*, implying *T. afroharzianum OE:SCf* strains could affect chitin synthesis.

Virulence proteins secreted by *B. cinerea* such as *BcCFEM*, *BcOAH*, *BcBMP1*, *BcPlc1*, and *BcPls1* were affected by *T. afroharzianum OE:SCf.* These genes were proven to modulate the virulence of *B. cinerea* [62–64]. Other research also showed that in the interaction zone of the aerial hyphae of *F. odoratissimum* and *T. guizhouense* contain proteolytic enzymes, hydrogen peroxide, and other metabolites [65, 66], those substances may also directly or indirectly participate the antagonistic process to *B. cinerea* by chimeric protein from engineered strains. It is well known that the great advantage of *Trichoderma*, based on accumulating knowledge of fungi-triggered plant immunity, is the induction of broad-spectrum pathogenic fungal resistance, which promises to be an effective and eco-friendly avenue to combat fungal diseases [5].

Overall, mycoparasitic interaction between the *Trichoderma* Sm1-chit42 engineered strain and *B. cinerea* involved the chemotaxis sensing, hyphal coiling, hydrophobicity modulation, cell wall adhesion, virulence reduction, and pathogen killing by ROS (Fig. 8). In conclusion, the chimeric protein SCf composed of Sm1 and Chit42, can enable synergistic action against *B. cinerea* as the following model.

### Electronic supplementary material

Below is the link to the electronic supplementary material.

**Additional file: Figure S1**. Construction of chimeric protein engineered strains of *T. afroharzianum*. (A) Sm1 and *Chit42* overlap fragments for chimeric protein and *TaSm1* and *MaChit42* overexpression vectors construction; (B) PCR verification of chimeric protein and *TaSm1* and *MaChit42* engineered strains by using hygromycin primer; (C) and (D) were PCR verification of chimeric protein and *TaSm1* and *MaChit42* engineered strains using by differential primer pairs (PC between trpC promoter and *Chit42*; CS between *Chi42* and Sm1; ST between Sm1 and trpC terminator; PS between trpC promoter and *Sm1*; SC between *Sm1* and *Chit42*; CT between *Chit42* and trpC terminator); (E) Southern blot analysis of chimeric protein and *TaSm1* and *MaChit42* engineered strains; (F) qPCR results of Sm1 gene expressing in *T. afroharzianum* with different culture medium (PDA and PD).

**Additional file: Figure S2**. *Sm1* gene expressing in the process of *T. afroharzianum* engineered strains interact with *B. cinerea*.

**Additional file: Figure S3**. Hydrophobicity modulation ability of *TaSm1, MaChi42*, and SCf expressing in *T. afroharzianum*. (A) Pictures and (B) box plot of a water droplet in the surface of *T. afroharzianum* wild-type (T30), *OE:TaSm1, OE:MaChi42*, and OE:SCf strains. Hydrophobicity of spores suspension of *T. afroharzianum* wild-type (T30), *OE:TaSm1, OE:MaChi42*, and *OE:SCf* strains in glass (C) and PET (D) slides.

**Additional file: Table S1** Primers used in this study.

## Data Availability

All data generated during this study are included in this published article.
