# Supplementary figures and images for: Fused expression of Sm1-Chit42 proteins for synergistic mycoparasitic response of Trichoderma afroharzianum on Botrytis cinerea
Source: Microb Cell Fact. 2023 Aug 17;22:156. doi: 10.1186/s12934-023-02151-w (PMC10433591; doi:10.1186/s12934-023-02151-w)

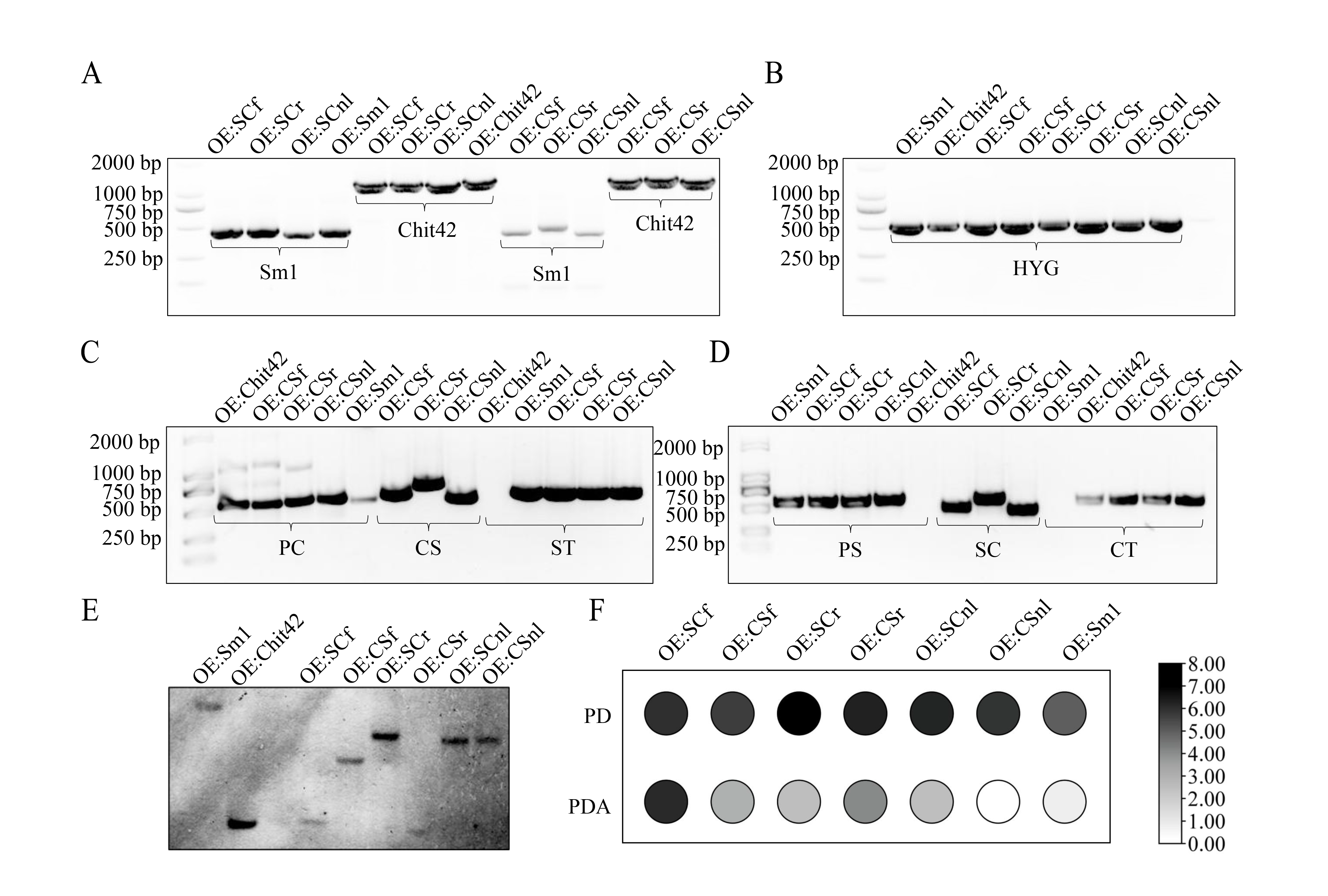

Supplement: Supplementary file 1 — Additional file: Figure S1. Construction of chimeric protein engineered strains of T. afroharzianum. (A) Sm1 and Chit42 overlap fragments for chimeric protein and TaSm1 and MaChit42 overexpression vectors construction; (B) PCR verification of chimeric protein and TaSm1 and MaChit42 engineered strains by using hygromycin primer; (C) and (D) were PCR verification of chimeric protein and TaSm1 and MaChit42 engineered strains using by differential primer pairs (PC between trpC promoter and Chit42; CS between Chi42 and Sm1; ST between Sm1 and trpC terminator; PS between trpC promoter and Sm1; SC between Sm1 and Chit42; CT between Chit42 and trpC terminator); (E) Southern blot analysis of chimeric protein and TaSm1 and MaChit42 engineered strains; (F) qPCR results of Sm1 gene expressing in T. afroharzianum with different culture medium (PDA and PD). [file 12934_2023_2151_MOESM1_ESM.png]

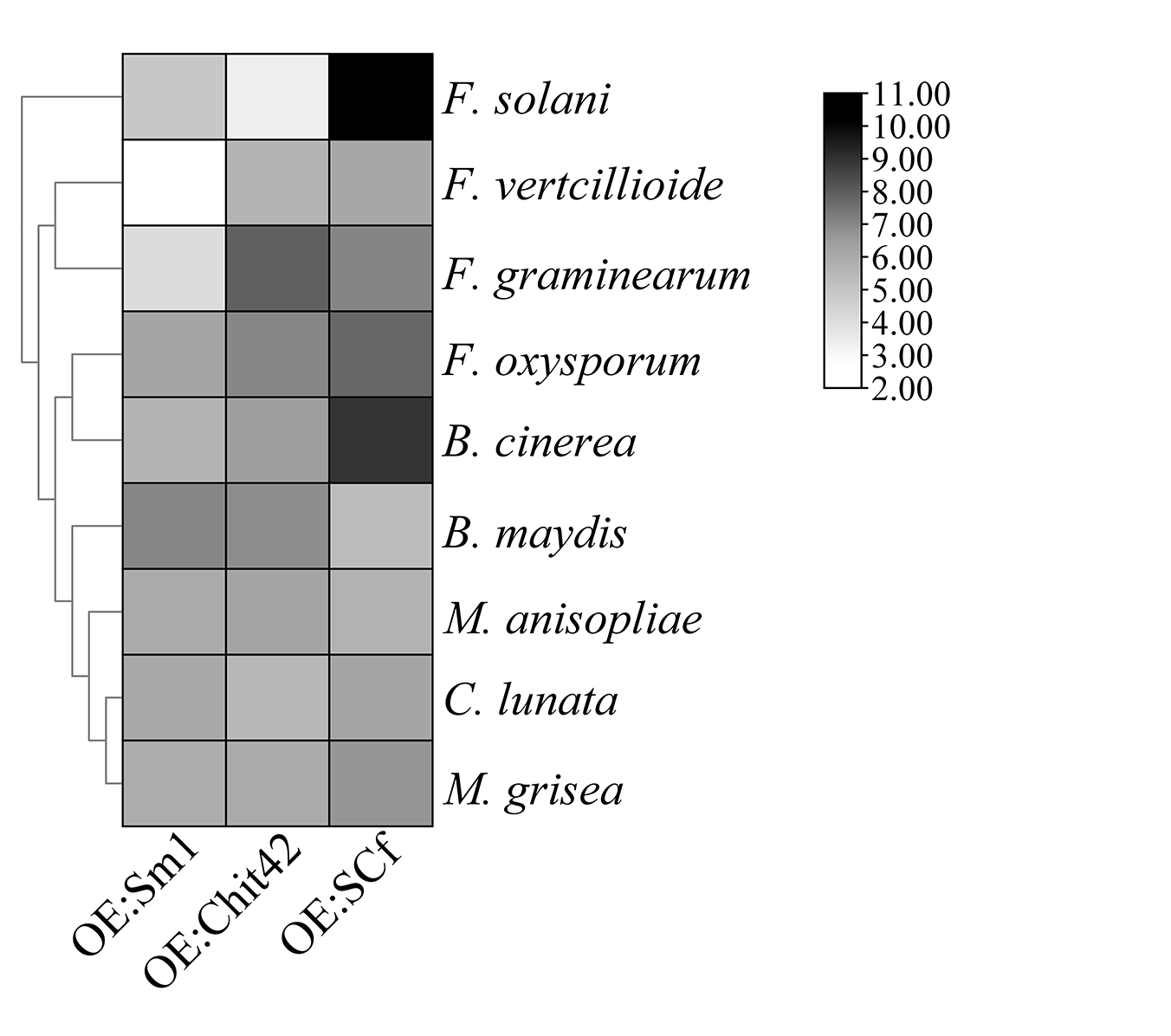

Supplement: Supplementary file 2 — Additional file: Figure S2. Sm1 gene expressing in the process of T. afroharzianum engineered strains interact with B. cinerea. [file 12934_2023_2151_MOESM2_ESM.png]

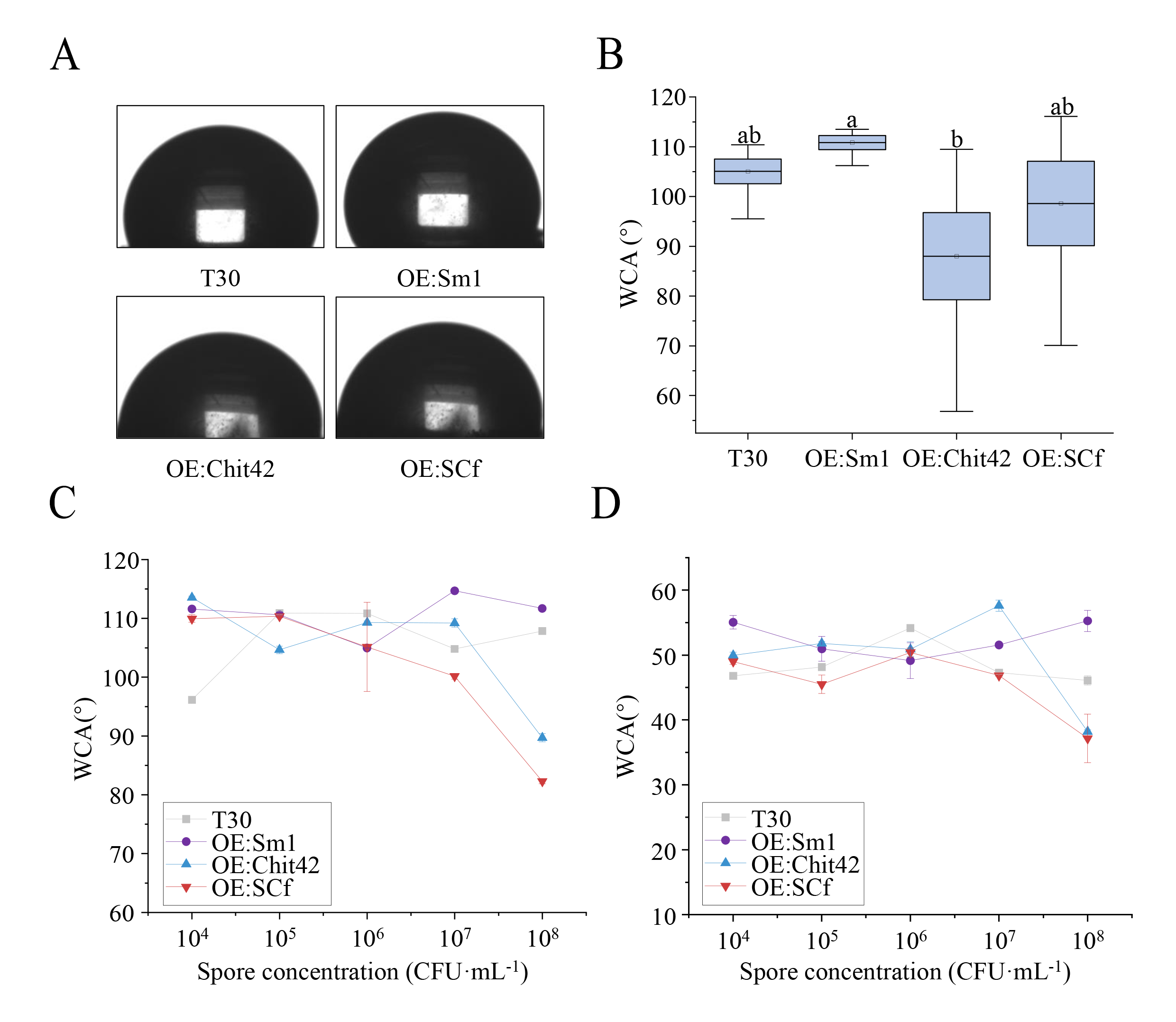

Supplement: Supplementary file 3 — Additional file: Figure S3. Hydrophobicity modulation ability of TaSm1, MaChi42, and SCf expressing in T. afroharzianum. (A) Pictures and (B) box plot of a water droplet in the surface of T. afroharzianum wild-type (T30), OE:TaSm1, OE:MaChi42, and OE:SCf strains. Hydrophobicity of spores suspension of T. afroharzianum wild-type (T30), OE:TaSm1, OE:MaChi42, and OE:SCf strains in glass (C) and PET (D) slides. [file 12934_2023_2151_MOESM3_ESM.png]
