# Supplementary material for: Fused expression of Sm1-Chit42 proteins for synergistic mycoparasitic response of Trichoderma afroharzianum on Botrytis cinerea
Source: Microb Cell Fact. 2023 Aug 17;22:156. doi: 10.1186/s12934-023-02151-w (PMC10433591; doi:10.1186/s12934-023-02151-w)
Supplement: Supplementary file 4 — Additional file: Table S1 Primers used in this study. [file 12934_2023_2151_MOESM4_ESM.docx]

**Table S1 Primers used in this study**

| **Primer** | **Sequence** | **Description** |
| --- | --- | --- |
| Sm1-F | 5’-aagcatcgatatgcaattgtccagcctcttcaag-3’ | Sm1 |
| Sm1f-R | 5’-gagtggcaagtgagcctccacctgagccacctcctgaacctccgcctgatccgccaccaagaccgcagttcttgacagc-3’ | Sm1-Chit42 with flexible linker |
| Sm1s-R | 5’-cggggcccagCTAGTGGTGATGGTGATGATGCTTGTCGTCATCATCAAGACCGCAGTTCTTGACA-3’ | Sm1 |
| Chit42s-F | 5’-aagcatcgatATGCCGTCGTTATTTGCTCAGT-3’ | Chit42 |
| Chit42-R | 5’-gttaagtggatccggggcccagctagtggtgatggtgatgatgcttgtcgtcatcatcagccatctgcttcctcatat-3’ | Chit42 |
| C42f-F | 5’-tggaggctcacttgccactcctgtatcagct-3’ | Sm1-Chit42 with flexible linker |
| PtrpC-R(PstI) | 5’-cggggcccagCTGCAGatcgatgcttgggtag-3’ | Sm1-Chit42 with rigid linker |
| TtrpC-F(PstI) | 5’-cgatCTGCAGctgggccccggatcc-3’ | Sm1-Chit42 with rigid linker |
| SLC-F | 5’-attctacccaagcatcgatCATGCAATTGTCCAGCCTCTTCAAG-3’ | Sm1-Chit42 with rigid linker |
| SLC-R | 5’-aagtggatccggggcccagCctagtggtgatggtgatgatgcttg-3’ | Sm1-Chit42 with rigid linker |
| Sm1r-R | 5’-CTTCGGCCTCCAATGCCTTTGCTGCGGCTTCCTTTGCAGCTGCCTCTTTGGCA GCAGCCTCTTTAGCAGCAGCTTCTGCCTCTAAAAGACCGCAGTTCTTGACAGC-3’ | Sm1-Chit42 with rigid linker |
| C42r-F | 5’-AAAGGCATTGGAGGCCGAAGCTGCCGCTAAAGAAGCAGCAGCAAAGGAAG  CTGCAGCTAAAGAAGCCGCCGCAAAGGCCCTTGAActtgccactcctgtatcagct-3’ | Sm1-Chit42 with rigid linker |
| C42nl-F | 5’-CGGTCTTcttgccactcctgtatcagct-3’ | Sm1-Chit42 without linker |
| Sm1nl-R | 5’-gtggcaagAAGACCGCAGTTCTTGACAGC-3’ | Sm1-Chit42 without linker |
| HPH-F | 5’-GGCGAAGAATCTCGTGCTTTCA-3’ | Hygromycin |
| HPH-R | 5’-CAGGACATTGTTGGAGCCGAAA-3’ | Hygromycin |
| mudeterPS-F | 5’-cctattttggtttagtcgtccaggc-3’ | Primer between trpC promoter and Sm1 |
| mudeterPS-R | 5’-CTGTACGACAGCTTCCAGCAG-3’ | Primer between trpC promoter and Sm1 |
| mudeterPC-F | 5’-ggaacgaggacattattatcatctgctg-3’ | Primer between trpC promoter and Chit42 |
| mudeterPC-R | 5’-ACGTCCACCCTCCAATAGACAAC-3’ | Primer between trpC promoter and Chit42 |
| mudeterSC-F | 5’-CGGCCACACCATCTACGTCCTGG-3’ | Primer between Sm1 and Chit42 |
| mudeterSC-R | 5’-cggctgggaagttcgttgaccacg-3’ | Primer between Sm1 and Chit42 |
| mudeterCT-F | 5’-gggcagcatgttttgggaggc-3’ | Primer between Chit42 and trpC teminator |
| mudeterCT-R | 5’-gtaggtctcttgacgaccgttgatct-3’ | Primer between Chit42 and trpC teminator |
| mudeterCS-F | 5’-CCTCAAGAGCAAGGGATTAGGGGGC-3’ | Primer between Chit42 and Sm1 |
| mudeterCS-R | 5’-GATCAACGGCCAGGACGTAGATGGT-3’ | Primer between Chit42 and Sm1 |
| mudeterST-F | 5’-AGATCCCTCGCTTCCCATACATC-3’ | Primer between Sm1 and trpC teminator |
| mudeterST-R | 5’-TCTGGAAGAGGTAAACCCGAAAC-3’ | Primer between Sm1 and trpC teminator |
| Sm1-F | 5’-ACTGCGGCATGGCTTGTGATATTC-3’ | Mycoparasitism related gene |
| Sm1-R | 5’-AACAAATACCACCGCAATCCGCAC-3’ | Mycoparasitism related gene |
| PRA1-F | 5’-ACCCTGGTCGGTGTTGTTTCTTGG-3’ | Mycoparasitism related gene |
| PRA1-R | 5’-TGATGAAGCTGACGTAGTTGCCCA-3’ | Mycoparasitism related gene |
| nag1-F | 5’-AGGCACCACAATCAAGGTTCCCTA-3’ | Mycoparasitism related gene |
| nag1-R | 5’-TTGGATCCATCCCAGTTGATTGAC-3’ | Mycoparasitism related gene |
| PM28-F | 5’-CTTCTCGGGTCTCAGGCAATCT-3’ | Mycoparasitism related gene |
| PM28-R | 5’-GTCAGTATAGACAGCCAGCTGGTT-3’ | Mycoparasitism related gene |
| PM14-F | 5’-CTATCAAGGCTTGACATAACGTAC-3’ | Mycoparasitism related gene |
| PM14-R | 5’-GATGGATGGATATGAAGGTAGAAG-3’ | Mycoparasitism related gene |
| exg1-F | 5’-GTTTGGATGTAAATGCGTTGCGCT-3’ | Mycoparasitism related gene |
| exg1-R | 5’-TCCATCATTATCCCAGGCCATCCA-3’ | Mycoparasitism related gene |
| sprT-F | 5’-AACTACGGCTCGGTTGTGGATATC-3’ | Mycoparasitism related gene |
| sprT-R | 5’-GGAGTTGCCATCGAGGTACCAGA-3’ | Mycoparasitism related gene |
| Lys-F | 5’-GTTATGCCAGTGATATTGCTAACC-3’ | Mycoparasitism related gene |
| Lys-R | 5’-GTACATACGGGATAAGAAAGTACG-3’ | Mycoparasitism related gene |
| papA-F | 5’-GACACAGTTACCTTCGGTGGTTTG-3’ | Mycoparasitism related gene |
| papA-R | 5’-GATCCACCGGACCAAAGCCGATAA-3’ | Mycoparasitism related gene |
| mutAW-F | 5’-ACAGACGACCATCAACCAGCATGT-3’ | Mycoparasitism related gene |
| mutAW-R | 5’-ATGACGGCGATACTCTTGGGTTTC-3’ | Mycoparasitism related gene |
| Lyase7-F | 5’-AATTCGGCGAGACTCAATCGTGGA-3’ | Mycoparasitism related gene |
| Lyase7-R | 5’-TGCCGTTGTAGTAAACCTCAGCGA-3’ | Mycoparasitism related gene |
| Chit42-F | 5’-ACTTTGGCCCAGACTTCCTCATCA-3’ | Mycoparasitism related gene |
| Chit42-R | 5’-TACCAGTCAATGTTGGAGGCCTGT-3’ | Mycoparasitism related gene |
| Tga1-F | 5’-TCCATCATTCTCTTCCTCAACAAG-3’ | Signal transduction gene |
| Tga1-R | 5’-ATAATCATCACCACCCTCGTAATC-3’ | Signal transduction gene |
| Tga2-F | 5’-GAGAACTACAGGTATCACAGAAAC-3’ | Signal transduction gene |
| Tga2-R | 5’-GACACTGATCGTAACCACTAATAG-3’ | Signal transduction gene |
| Tga3-F | 5’-CTCAACCTATATCCACATCTTACC-3’ | Signal transduction gene |
| Tga3-R | 5’-CCTTGAGTGCGTTATTCAGTATAG-3’ | Signal transduction gene |
| Ime2-F | 5’-CTCCAGCAGCAACAACTATA-3’ | Signal transduction gene |
| Ime2-R | 5’-AGAAGAAGCTCTCCTTGTGA-3’ | Signal transduction gene |
| Kss1-F | 5’-CATCATCTCCATTCTTGATATCCAG-3’ | Signal transduction gene |
| Kss1-R | 5’-CGCAGAGTCTGATAGATAAAGTAC-3’ | Signal transduction gene |
| Slt2-F | 5’-CTATCAAGAAGGTCACCAATGTCT-3’ | Signal transduction gene |
| Slt2-R | 5’-CTAGGAATGTCCATATCATAGAGG-3’ | Signal transduction gene |
| Hog1-F | 5’-CTCTCTCAGCGATATCTTCATCTC-3’ | Signal transduction gene |
| Hog1-R | 5’-GATCTGATAGAGGAAGTACTGGAT-3’ | Signal transduction gene |
| qTef1-F | 5’-TACAAGATCGGTGGTATTGGAACA-3’ | Housekeeping gene |
| qTef1-R | 5’-AGCTGCTCGTGGTGCATCTC-3’ | Housekeeping gene |
| BcBMP1-q-F | 5’-TCTTTCAATGTCAGCGAGCAA-3’ | *B. cinerea* virulence gene |
| BcBMP1-q-R | 5’-TGCAAAGCTGAGCAGACAACA-3’ | *B. cinerea* virulence gene |
| BcPLC1-q-F | 5’-TCCCGCAGGACTCGATAACT-3’ | *B. cinerea* virulence gene |
| BcPLC1-q-R | 5’-TATGGCTTCCACTCGGGTTT-3’ | *B. cinerea* virulence gene |
| BcPLS1-q-F | 5’-CGCCTTCCTCATCTCCATTC-3’ | *B. cinerea* virulence gene |
| BcPLS1-q-R | 5’-CAACGACGAAGAAGCCATGAA-3’ | *B. cinerea* virulence gene |
| BcActin-F | 5’-CATGGCTGGTCGTGATTTGA-3’ | *B. cinerea* virulence gene |
| BcActin-R | 5’-GAGGATTGACTGGCGGTTTG-3’ | *B. cinerea* virulence gene |
| BcCFEM-F | 5’-AAGAGGAGGATGTGGGGTCA-3’ | *B. cinerea* virulence gene |
| BcCFEM-R | 5’-CTAGCACATCGACGTCCTCC-3’ | *B. cinerea* virulence gene |
